# The Histological and Clinical Changes Induced by Radium in Carcinoma and Sarcoma

**Published:** 1914-11

**Authors:** 


**The Histological and Clinical Changes Induced by Radium in Carcinoma and Sarcoma.** Dr. W. H. B. Aikins and K. M. B. Simon, M. B., L. M. C. C., Toronto. "Dominion Medical Monthly," Sept. 1914.

In sarcomata the retrogression takes place according to the following law:

1. The size of the body and of the nucleus of the large cells decreases.

2. As they shrink the neoplastic elements elongate, the shape of the nucleus becomes regular, and they eventually assume the form of large embryonic connective tissue cells, forming into a celled mass similar to that of a true fibroma. Thus we may emphasize the fact that sarcomata are transformed by radium into a tissue analagous to that of a fibroma with myxomatous changes.

As regards epitheliomata and carcinomata, under the influence of the radium rays, the following change takes place:

1. The cells gradually diminish in size and staining properties.

2. This atrophy corresponds not to the metamorphosis of these definite formed elements, but to their destruction as shown by keratinization or absorption.

3. The epitheliomatous cells disappear either by means of progressive absorption of protoplasm and nuclei through the leucocytic infiltration or by a sort of granular degeneration.

The other processes associated with the development of every epithelial tumor are arrested, while vascular connective tissue is organized according to the method just described.

4. As proof that the changes initiated by radium in the tumors are such as to lead to immunity, great importance must be attached to the cellular infiltration, first leucocytic, then later a round celled infiltration. It has been recently shown these require different reactions of the tissue for their function, hence radium must affect the blood.[9] These infiltrations have always been noted in all cases of experimental transplantation of malignant cells, and always accompany the cases in which the animal becomes immune and the tumor disintegrated[8].

